# The detection of mixed tuberculosis infections using culture filtrate and resuscitation promoting factor deficient filtrate

**DOI:** 10.3389/fcimb.2022.1072073

**Published:** 2022-11-24

**Authors:** Melissa D. Chengalroyen, Germar M. Beukes, Kennedy Otwombe, Bhavna G. Gordhan, Neil Martinson, Bavesh Kana

**Affiliations:** ^1^ National Health Laboratory Service, DST/NRF Centre of Excellence for Biomedical TB Research, University of the Witwatersrand, Johannesburg, South Africa; ^2^ Perinatal HIV Research Unit, Faculty of Health Sciences, University of the Witwatersrand, Johannesburg, South Africa; ^3^ Center for TB Research, Johns Hopkins University, Baltimore, MD, United States

**Keywords:** resuscitation promoting factor, *Mycobacterium tuberculosis*, mixed infection, differentially culturable tubercle bacteria, most probable number assay

## Abstract

Tuberculosis (TB) infected individuals harbor a heterogenous population of differentially culturable tubercle bacilli (DCTB). Herein, we describe how DCTB assays using culture filtrate either containing or deficient in resuscitation promoting factors can uncover mixed infections. We demonstrate that *Mycobacterium tuberculosis* (*Mtb*) strain genotypes can be separated in DCTB assays based on their selective requirement for growth stimulatory factors. Beijing mixed infections appear to be associated with a higher bacterial load and reduced reliance on growth stimulatory factors. These data have important implications for identifying mixed infections and hetero-resistance, which in turn can affect selection of treatment regimen and establishment of transmission links.

## Introduction

*Mycobacterium tuberculosis* (*Mtb*), the causative agent of tuberculosis (TB), is a pathogen whose successful adaptation to the human host has been paramount to its colonization of one-fourth of the globe’s population (Cohen et al., 2019). Challenges contributing to the control of TB disease include a strong association with HIV infection in certain endemic settings, evolution of multi-drug resistance, infection with multiple *Mtb* strains and the recent long term negative impact of the COVID-19 pandemic (Wingfield et al., 2021).

Traditionally, the commonly accepted modes of TB disease acquisition were associated with primary infection or reactivation with a single strain (Milburn, 2001; Ai et al., 2016). However, increased reports of mixed strain infections, particularly in retreatment cases (Warren et al., 2004) or in high TB incidence settings (Verver et al., 2005) have been documented. These are most likely acquired by simultaneous infection with more than one *Mtb* strain or endogenous reactivation and subsequent secondary infection with a distinct strain (Perez-Lago et al., 2015). Patients with mixed *Mtb* strains, particularly with different drug resistance profiles, may not respond well to standard anti-TB chemotherapy. As an example, the challenge of treating mixed infections was demonstrated in a case study where the treatment regimen cleared one strain but was ineffective against the second strain, resulting in prolonged persistence of that isolate (Abascal et al., 2021). *Mtb* mixed infections ultimately predict a higher probability of poor outcomes (Shin et al., 2018).

The ability to detect mixed infections is influenced by numerous factors. These include the timing of sputum sampling relative to treatment, the specific lesions open to the airway at the time of sampling, the specimen handling process which could lead to a reduction in prevalence of the minority variant, sensitivity of the assay being used and retreatment of TB disease (Cohen et al., 2012). In addition, broader aspects such as socio-economic conditions, TB-incidence rate (Richardson et al., 2002), host genetic predispositions and socio-demographics also influence the frequency of *Mtb* mixed infections. Considering the interplay of these complicated factors, its unsurprising that the prevalence of *Mtb* mixed infections documented in various settings is highly variable, ranging from 1.3% (2/160), 2.3% (3/131), 14.7% (15/102), 3% (14/466), 26.6% (20/75), 0.01% (9/703) to 19% (35/186) (Richardson et al., 2002; Warren et al., 2004; Mallard et al., 2010; Wang et al., 2010; Navarro et al., 2011; Hajimiri et al., 2016; Mustafa et al., 2016). In addition, another study observed an incidence of 51% (26/51) mixed infections, when comparing blood against sputum samples (Ssengooba et al., 2015).

Molecular typing methods used to distinguish mixed *Mtb* strains include 24 loci mycobacterial interspersed repetitive unit variable tandem repeat typing (MIRU-VNTR), spoligotyping, whole genome sequencing (WGS) or IS6110-RFLP (Das et al., 2004). These methods are useful, however most depend on the visualization of low intensity bands (corresponding to the minority strain) against prominent bands of the majority strain, which can be subjective. For WGS, conventional culturing of samples in liquid media often favors the growth of the predominant strain over the minority variant, thus minimizing the ability to detect multiple strain infections (Martin et al., 2010). Also, for most techniques, samples must constitute at least 10% of the minority strain for detection (de Boer et al., 2000), while for WGS, the minority population must be > 10% (Sobkowiak et al., 2018). Warren et al. (2004) developed a simple rapid PCR-based assay to detect mixed Beijing/non-Beijing infections for a more accurate measure of the proportion of individuals harboring multiple strains (Wang et al., 2010). To our knowledge no such method has been developed to distinguish mixed genotypes within the same lineage. Advancements in the identification of mixed infections will reconcile the true percentage of individuals infected with more than one *Mtb* strain, facilitating optimal treatment.

Our previous findings indicated that sputum from TB infected individuals contains Differentially Culturable Tubercle Bacilli (DCTB), a population of bacteria which have a requirement for growth stimulatory factors in culture filtrate (CF) to either recover from a damaged state or reawaken from a state that precludes growth on standard laboratory media (Chengalroyen et al., 2016). Within CF, the presence of resuscitation promoting factors (Rpfs), a group of growth stimulatory enzymes, has been associated with recovery of DCTB (Mukamolova et al., 2009; Chengalroyen et al., 2016). However, DCTB populations have also been shown to grow in CF deficient of Rpfs, suggesting that other molecules in CF also have a growth stimulatory effect (Chengalroyen et al., 2016; Gordhan et al., 2021). The presence of these differentially culturable populations suggests that use of routine culture media only allows for a subset of bacteria to emerge, possibly limiting the detection of all genotypes present in a sputum sample. In this study we used Most Probable Number (MPN) assays with growth factor supplementation to identify mixed strains in sputum obtained from TB infected individuals. Spoligotyping was used to identify the different strains, if any, from each participant. We demonstrate that this approach allows for the detection of mixed strain infections.

## Materials and methods

### Study population

This study was approved by the Human Research Ethics Committee of the University of Witwatersrand (M110532). One-hundred and ten drug sensitive TB infected patients from Soweto in Johannesburg, South Africa were enrolled. Participants provided written consent and were requested to provide a sputum sample prior to treatment initiation. Patients were older than 18 years with either a GeneXpert or smear positive TB result. For more comprehensive detail of the patient cohort, clinical characteristics and methodology used which includes sputum sample preparation, bacterial culturing and quality control checks, the reader is referred to (Chengalroyen et al., 2016).

### MPN assay

**Figure 1:**
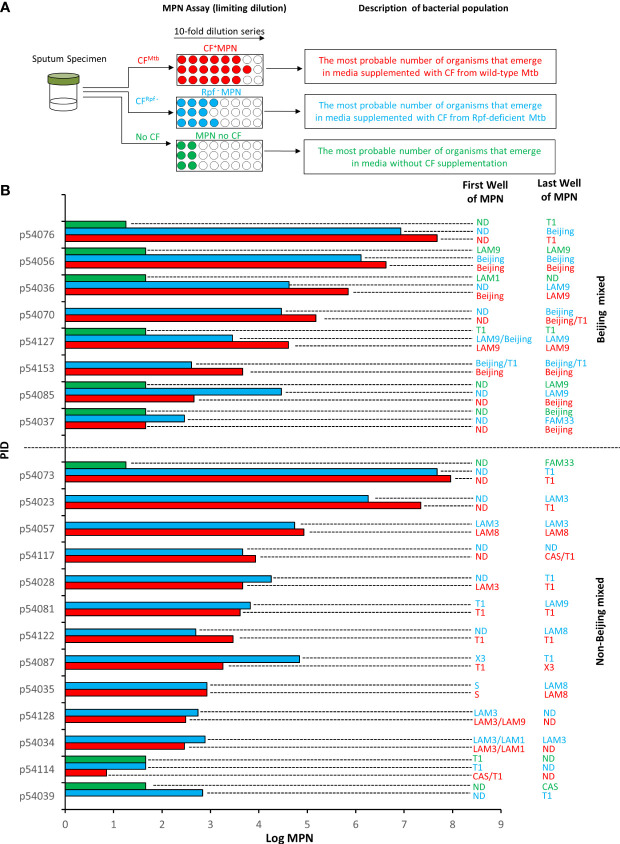
Mycobacterial family strains identified in Most Probable Number (MPN) assays. **(A)** Shown is a schematic representation of the experimental workflow. Sputum specimens were decontaminated and the same specimen was subjected to three MPN assays in the presence of culture filtrate (CF) from wild type *M. tuberculosis* (CF^Mtb^), CF from an Rpf-deficient mutant of *M. tuberculosis* (CF^Rpf-^) and no CF. The populations detected in this experimental set up are shown. **(B)** Shown is the strain identified in the first well and last well of CF^+^MPN (red), Rpf ^–^ MPN (blue) and MPN no CF (green) assays. The absence of a MPN value reflects no growth under that condition. Wells in which Beijing strains were detected with another strain “Beijing Mixed” (top set) are delineated from those with only Non-Beijing strains “Non-Beijing mixed” (lower set) by the horizontal dashed line. Spoligotyping was used to genotype isolates present in the MPN wells. ND, not determined.

The MPN assay was conducted as previously described (Chengalroyen et al., 2016). This technique involves performing a 10-fold serial dilution of bacilli and uses the Poisson distribution to estimate the number of viable cells based on the dilutions which show the presence vs the absence of visible growth. The experimental outline is given in **Figure 1A**. Briefly, sputum samples were decontaminated and resuspended in 7H9 media. Thereafter, the cells were dispersed by vortexing with glass beads. To determine the MPN, the sputum sample was added to the first well of a 48-well microtiter plate and subsequently diluted 10-fold to the end of the plate. The media was supplemented in a 1:1 ratio with i) CF harvested from *Mtb* H37Rv grown in 7H9 media, ii) Rpf **^–^** CF, CF harvested from *Mtb* BG1, a quintuple mutant deficient in all five *rpf*-encoding genes (Kana et al., 2008) and iii) standard 7H9 media as a control. Following the dilution, the microtiter plates were incubated for six weeks. Thereafter, the growth pattern in wells was recorded and used to determine bacterial count. The first and last well of the microtitre plate showing visible growth for each condition was harvested. An aliquot was used to extract DNA from the bacteria by heat killing at 95°C for 30 minutes, in preparation for spoligotyping. Additionally, in a select case (participant 54034), bacilli from the first and last wells were spread onto 7H10 plates to isolate individual colonies. After 3-4 weeks of incubation, individual colonies were picked, followed by heat killing at 95°C for 30 minutes to extract DNA in preparation for spoligotyping.

### Spoligotyping

Spoligotyping was performed as per the manufacturer’s instruction using a kit (Ocimum Biosolutions) to amplify IS6110 regions. Amplified IS6110 products were hybridized to 43 immobilized oligonucleotides on a membrane representing unique spacer regions. Hybridized DNA was detected by chemiluminescence (Amersham Biosciences) and exposed to X-ray film. Mycobacterial strain identification was conducted by comparison of the resulting pattern to the SPOTCLUST database (https://tbinsight.cs.rpi.edu/run_spotclust.html).

### Data analysis

Specimens were categorized as harboring either *Mtb* Beijing mixed (simultaneous presence of a Beijing and non-Beijing strain) or *Mtb* non-Beijing mixed strains (simultaneous presence of two non-Beijing strains). Interquartile ranges were determined for each variable using the Kruskal-Wallis Test.

## Results

**Figure 2:**
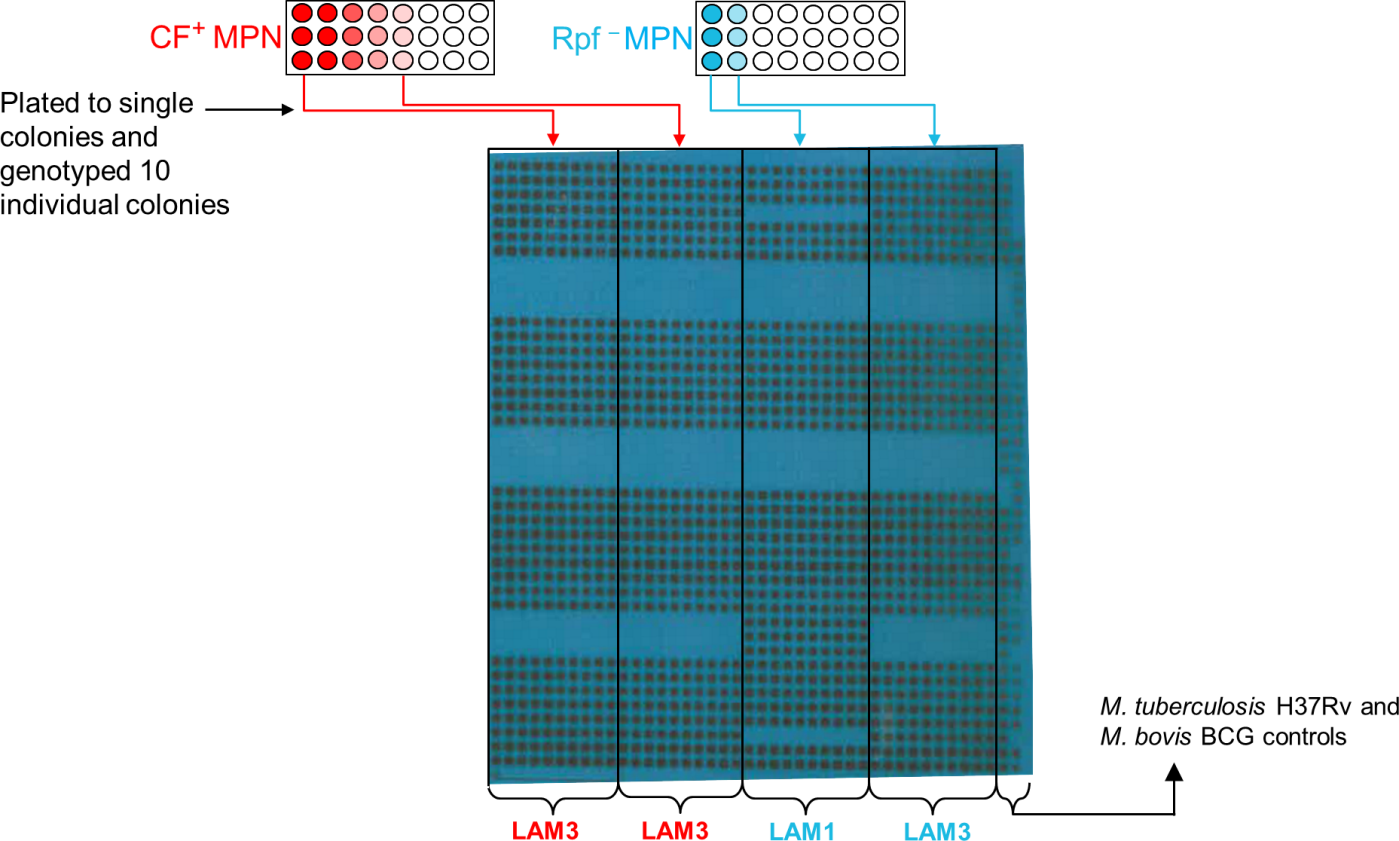
Detection of different strain types in MPN wells. Aliquots from the MPN wells from participant 54034 were plated to single colonies and 10 individual colonies were genotyped. Shown is the blot from the spoligotyping of these colonies.

Each sputum sample in our collection was subjected to three MPN assays supplemented with CF, Rpf-deficient CF and no CF (**Figure 1A**). The patterns of positive and negative growth were used to determine the MPN. The bacterial yield from these three assays for the specimens reported in this study are given in **Figure 1B**. From these assays, the first and last wells from MPN plates were sampled and where possible, a spoligotype was determined. Of the 102 spoligotype profiles obtained from our collection of specimens, 21% (21/102) carried mixed infections, as observed by the detection of a different strain in i) the culture obtained from the first well versus the last well in the CF^+^ MPN or Rpf **^–^** MPN condition, or ii) in any well of the CF^+^ MPN when compared to either the Rpf **^–^** CF or media control (MPN no CF) wells, **Figure 1B**. To further confirm the presence of mixed strains, the bacteria from the first and last MPN well, for a randomly selected isolate, was plated to single colonies and the individual colonies were spoligotyped. Two distinct strains emerged in the MPN assays (**Figure 2**). Spoligotyping of the entire bacterial population from the first well of the MPN plates identified the LAM9 strain however, the single colonies isolated from the culture showed the presence of a mixture of LAM1 and LAM3 suggesting that the LAM9 spoligotype pattern observed in the original analysis of the culture from the MPN well was the result of LAM1 and LAM3 superimposed spacer regions (**Figure 2**). These data indicate that the LAM3 strain outcompeted the LAM1 strain in the presence of CF containing Rpfs while the absence of Rpfs in CF exhibited a selective advantage for the LAM1 strain (Rpf **^–^** MPN first well). It is likely however that LAM1 was diluted out in the last Rpf **^–^** MPN well since LAM3 re-emerged. These observations point to a complex interplay between infecting strains whereby particular strains emerge differentially in the presence or absence of CF or Rpf **^–^** CF.

To expand on this, we searched for correlations among pathology, immunology, diagnostics and DCTB profile relative to the 8 Beijing-mixed and 13 non-Beijing mixed infections in our cohort. Notably, the small sample size was a limitation, precluding extensive analysis and determination of statistical significance. Nevertheless, we observed that the Beijing mixed strains grew slightly better in the presence of CF containing Rpfs compared to the absence of Rpfs (MPN of 4.9 vs 3.5). We also noted that individuals harboring Beijing-mixed infections carried a higher quantum of CF^+^ MPN and Rpf **^–^** MPN organisms (MPN 4.9 and 4.5) compared to non-Beijing mixed carriers (MPN 3.5 and 3.7) (**Table 1**). Additionally, the propensity for spontaneous reactivation (without the requirement for additional growth factors), inferred from the MPN no CF media control, was observed strictly within Beijing mixed strains (**Table 1**). Furthermore, colony forming units were higher from Beijing mixed infections when compared to non-Beijing mixed carriers (640.0 vs 0.0) (**Table 1**). These observations should be explored in a larger cohort.

**Table 1:** Demographics and clinical characteristics of patient participants categorized as carriers of either Beijing or non-Beijing *Mtb* mixed infection.

Variable	Overall (n=21)	Beijing mixed (n=8)	Non-beijing mixed (n=13)
**Demographics**			
Female (%)	9.0 (42.9)	3.0 (37.5)	6.0 (46.2)
Male (%)	12.0 (57.1)	5.0 (62.5)	7.0 (53.8)
**Median age in years (IQR)**	39.0 (32.0-44.0)	36.0 (29.5-42.5)	40.0 (34.0-44.0)
Minimum, maximum	22.0-69.0	24.0-69.0	22.0-64.0
**BMI (IQR) in kg/m^2^**			
Underweight (%)	6.0 (28.6)	3.0 (37.5)	3.0 (23.1)
Normal (%)	12.0 (57.1)	3.0 (37.5)	9.0 (69.2)
Overweight (%)	3.0 (14.3)	2.0 (25.0)	1.0 (7.7)
Median BMI (IQR)	20.3 (17.7-23.6)	18.8 (16.9-24.2)	20.5 (18.8-23.6)
Minimum, maximum	10.1-30.5	10.1-30.5	16.9-26.0
Lung pathology*			
No cavity (%)	2.0 (11.8)	1.0 (14.3)	1.0 (10.0)
Cavity (%)	15.0 (88.2)	6.0 (85.7)	9.0 (90.0)
**Extent of disease** ^†^			
Limited (%)	3.0 (14.3)	2.0 (25.0)	1.0 (7.7)
Moderate (%)	9.0 (42.9)	2.0 (25.0)	7.0 (53.8)
Extensive (%)	5.0 (23.8)	3.0 (37.5)	2.0 (15.4)
Unknown (%)	4.0 (19.0)	1.0 (12.5)	3.0 (23.1)
**Patient immunology** ^‡^			
**HIV status**			
Negative (%)	13.0 (61.9)	5.0 (62.5)	8.0 (61.5)
Positive (%)	8.0 (38.1)	3.0 (37.5)	5.0 (38.5)
**Median CD4 count (IQR) cells/mm^3^ (only HIV infected)**	211.0 (116.0-300.0) n = 7.0	211.0 (34.0-223.0) n = 3.0	219.5 (127.5-357.0) n = 4.0
Minimum, maximum	34.0-414.0	34.0-223.0	116.0-414.0
HAART treatment (%) ^∏^	1.0 (12.5)	0.0 (0.0)	1.0 (20.0)
**Conventional TB diagnosis**			
Smear Grade negative (%)	5.0 (23.8)	1.0 (12.5)	4.0 (30.8)
Smear Grade positive (%)	16.0 (76.2)	7.0 (87.5)	9.0 (69.2)
GeneXpert result ^**§^			
High (%)	2.0 (12.5)	1.0 (14.3)	1.0 (11.1)
Medium (%)	5.0 (31.3)	3.0 (42.9)	2.0 (22.2)
Low (%)	6.0 (37.5)	2.0 (28.6)	4.0 (44.4)
None (%)	3.0 (18.8)	1.0 (14.3)	2.0 (22.2)
Median GeneXpert cycle threshold (IQR)	18.7 (15.5-23.6) n = 16.0	18.2 (15.3-23.5) n =7.0	22.6 (15.8-23.6) n = 9.0
Minimum, maximum	0.0-28.6	0.0-23.6	0.0-28.6
Median MGIT days to positivity (IQR) ^††^	12.0 (10.5-21.0) n = 20.0	12.0 (10.0-13.0) n = 7.0	12.0 (11.0-29.0) n = 13.0
Minimum, maximum	5.0-42.0	8.0-22.0	5.0-42.0
**Most probable number [MPN] (IQR)**			
Log median CF^+^ MPN	3.7 (2.7-5.2)	4.9 (3.2-6.2)	3.5 (2.5-3.9)
Minimum, maximum	0.0-8.0	1.7-7.7	0.0-8.0
Log median Rpf ^–^ MPN	3.8 (2.8-4.7)	4.5 (3.0-5.4)	3.7 (2.8-4.7)
Minimum, maximum	0.0-7.7	2.5-6.9	0.0-7.7
Log median MPN no CF	0.0 (0.0-1.3)	1.5 (0.0-1.7)	0.0 (0.0-0.0)
Minimum, maximum	0.0-1.7	0.0-1.7	0.0-1.7
Median MPN days to positivity (IQR)	14.0 (14.0-21.0)	14.0 (14.0–17.5)	14.0 (14.0-21.0)
Minimum, maximum	7.0-49.0	7.0-35.0	7.0-49.0
Median Colony forming units [CFU] (IQR)	50.0 (0.0-600.0)	640.0 (32.5-15750.0)	0.0 (0.0-70.0)
Minimum, maximum	0.0-32500	0.0-32500	0.0-29500

The sample size was too small to test statistical assumptions, hence the p-value was not calculated. IQR represents the interquartile range and bracketed numbers represent the percentage IQR.

^*^4 participants did not have cavitation data.

^†^4 participants did not have disease extent data.

^‡^1 participant did not have CD4 count data.

^§^5 participants had no GeneXpert data.

^∏^only 1 participant was on HAART.

^**^5 participants did not have GeneXpert data.

^††^1 participant had missing MGIT data.

## Discussion

The classification of TB as a binary disease of either active versus latent infection has evolved, with the current outlook suggesting that TB infection exists as a spectrum (Lin and Flynn, 2018) ranging from incipient or subclinical disease with/without symptoms to active disease (Migliori et al., 2021). We posit that underlying these disease states are heterogenous populations of bacteria, including DCTB that appear to be modulated by the host immune response (Chengalroyen et al., 2016). These have been identified in the sputum of drug sensitive (Mukamolova et al., 2010; Chengalroyen et al., 2016; McAulay et al., 2018) and drug resistant TB infected patients (Saito et al., 2017; Zainabadi et al., 2021; Gordhan et al., 2022) in addition to being present at extrapulmonary sites of infection (Rosser et al., 2018). In this study, using DCTB assays, we identified mixed infections at a prevalence of 21% in sputum samples from drug sensitive participants. Strains displayed growth stimulation/suppression in response to unidentified selective factors in CF and could be segregated using the MPN assay. Plazzotta et al. (2015) established a mathematical model showing that the initial quantity of a minor variant and its growth rate are the main factors which affect its detection, predicting that if the minority strain is present in < 3% of the sample, it will not be detected, thus misclassifying the patient with a single strain infection. Notably, in the MPN assay, dilution of the majority strain and favored growth of the minority strain may alleviate these limitations. Using this method, same family genotype mixed infections could be detected. However, to be able to distinguish modern and ancient Beijing mixed infections, this assay would need to be conducted in conjunction with a more sensitive genotyping method such as MIRU-VNTR or WGS.

The Beijing *Mtb* lineage is characterized by enhanced virulence, higher transmission rates and an increased association with drug resistance (Hanekom et al., 2011). Notably in this study, patients with Beijing mixed infection had a higher bacterial load as estimated by CFUs and MPN (**Table 1**) and did not require stimulatory factors for efficient growth. It is possible that the Beijing genotype exploits or suppresses the non-Beijing isolate to gain a growth advantage (von Groll et al., 2010; Wang et al., 2010), although the mechanism/s underlying this remains to be determined. Further analysis of our patient cohort indicted that no host parameters were specifically associated with Beijing or non-Beijing DCTB mixed infection (**Table 1**).

Multiple infections can lead to discordant drug susceptibility profiles (Mei et al., 2015) with instances of patients carrying a drug sensitive and drug resistant strain (Baldeviano-Vidalon et al., 2005). Also, in mixed Beijing infections there exists a higher probability of resistance to at least one drug (Huang et al., 2010). The identification of dual/multiple strain infection could allow for a change in treatment regimen, a higher antibiotic dosage and lengthened treatment to improve outcomes. The separation of different *Mtb* genotypes with this assay premises a phenotypic and/or metabolic disparity between the strains. Consequently, this could be a gateway to better understanding competition, suppression and mutualism-based interactions between different *Mtb* genotypes.

## Data availability statement

The original contributions presented in the study are included in the article/supplementary material. Further inquiries can be directed to the corresponding author.

## Ethics statement

The studies involving human participants were reviewed and approved by Human Research Ethics Committee, University of the Witwatersrand. The patients/participants provided their written informed consent to participate in this study.

## Author contributions

MC and GB performed the experiments. KO performed the statistical analysis. NM recruited patients for the study and assembled appropriate patient data. BG and BK designed the experiments. All authors contributed to the article and approved the submitted version.

## Funding

This study was supported by the National Institutes of Health (U01 AI069453-07), National Research Foundation of South Africa, South African Medical Research Council, Centre for Aids Prevention Research in South Africa and the Howard Hughes Medical Institute.

## Conflict of interest

The authors declare that the research was conducted in the absence of any commercial or financial relationships that could be construed as a potential conflict of interest.

## Publisher’s note

All claims expressed in this article are solely those of the authors and do not necessarily represent those of their affiliated organizations, or those of the publisher, the editors and the reviewers. Any product that may be evaluated in this article, or claim that may be made by its manufacturer, is not guaranteed or endorsed by the publisher.
